# Impaired peripheral oxygen delivery during submaximal exercise in adults with long COVID

**DOI:** 10.14814/phy2.70873

**Published:** 2026-04-23

**Authors:** Callum Thomas, Ruth E. Ashton, Rebecca Owen, Ethan McNeil‐Angopa, Jack Carr, Thomas Bewick, Mark A. Faghy

**Affiliations:** ^1^ School of Health, Sport and Rehabilitation University of Derby Derby UK; ^2^ Research Centre for Physical Activity, Sport and Exercise Sciences (PASES) Coventry University Coventry UK; ^3^ Department of Respiratory Medicine University Hospitals of Derby and Burton NHS Foundation Trust Derby UK; ^4^ School of Sport, Exercise and Health Sciences Loughborough University Loughborough UK

**Keywords:** COVID‐19, CPET, hypoxia, long COVID, NIRS

## Abstract

Long COVID (LC) is a multisystem condition that is linked to distinct pathologies including viral persistence, immunological dysfunction, endothelial damage, and mitochondrial dysfunction. To date, limited research has assessed peripheral tissue hypoxia to better understand LC symptom exacerbation. Forty‐six people with LC and 10 controls (CON) completed two submaximal cardiopulmonary exercise tests (CPETs), separated by 24‐h. Near‐infrared spectroscopy (NIRS)‐derived signals from the left gastrocnemius muscle were continuously monitored before, during, and after 2‐day incremental CPET. CPET outcomes demonstrated impaired physical function on day 2 compared with day 1 for the LC cohort at rest and VT1. LC tissue saturation index (TSI%) remained elevated above rest for a shorter duration of exercise compared to CON on day 1 (2nd minute vs. 5th minute). On day 2, this response worsened for LC (Rest vs. 1st exercise minute: 63 ± 5% vs. 65 ± 5%; *p* < 0.05); meanwhile, CON exhibited sustained TSI% elevation throughout exercise above rest (Rest vs. 12th exercise minute: 62 ± 5% vs. 67 ± 4%; *p* < 0.05). LC TSI% remained elevated above rest for a shorter duration of exercise compared to CON, worsening for LC on day 2. LC showed rapid normalization of TSI%, suggesting impaired muscle oxygenation and recovery during repeated exercise.

## 1 INTRODUCTION

Long COVID (LC) is a continuation or emergence of symptoms following an infection with SARS‐CoV‐2 that can have profound impacts on daily functioning and quality of life (National Academies of Sciences, Engineering, and Medicine, 2024). LC symptoms are prone to fluctuate and exacerbation, known as post‐exertional symptom exacerbation (PESE) from physical, cognitive, and psychosocial stimuli (Davenport et al., 2023; Thaweethai et al., 2023). Pathologies of LC are well documented and include viral persistence, immunological dysfunction, endothelial and platelet dysfunction, mitochondrial dysfunction, and prolonged respiratory complications (Faghy et al., In Press). Heightened, persistent inflammation underlies many of these complications, and this proinflammatory state may well contribute to both acute and chronic symptoms of tissue hypoxia (Østergaard, 2021).

A hallmark observation of acute SARS‐CoV‐2 infection is the onset of a COVID‐19 associated cytokine storm which is characterized by the excessive release of pro‐ and anti‐inflammatory cytokines (e.g., Interleukin‐1 (IL‐1, IL‐2, IL‐6, IL‐15), tumor necrosis factor‐alpha (TNF‐α), and interferon‐gamma (IFN‐γ)) and activation of immune cells (Arena et al., 2022; Hu et al., 2021; Yang et al., 2021). Disproportionate, acute inflammation following a SARS‐CoV‐2 infection increases vascular permeability of the alveolar‐capillary barrier, reduces lung compliance and size of aerated tissue, compromises gas exchange and causes hypoxemia (Bellani et al., 2016). This process may be further exacerbated by endothelial damage, microvascular dysfunction, and persistent microthrombi, leading to ventilation‐perfusion mismatch (Donina, 2022) which is a catalyst for several key pathologies associated with LC. Furthermore, fibrotic changes in lung tissue can limit alveolar expansion, while chronic low‐grade inflammation perpetuates vascular and structural alterations. Additionally, mitochondrial dysfunction in skeletal muscle and autonomic dysregulation may impair peripheral oxygen utilization and impact cardiovascular responses during exercise, contributing to persistent symptoms in LC (Colosio et al., 2023; Mooren et al., 2023).

Reduced exercise capacity in people living with LC has been reported by several single‐day CPET protocols demonstrating impairment in cardiorespiratory and muscular systems (Appelman et al., 2024; Colosio et al., 2023; Durstenfeld et al., 2022). As part of this dysfunction, peripheral limitations have been partly attributed toward test outcomes; for example, Colosio et al. report limited exercise capacity in people with LC that was mainly due to lower muscle oxidative capacity of the vastus lateralis, and substantial reductions of mitochondrial function biomarkers (e.g., citrate synthase) compared to a control group. Near‐infrared Spectroscopy (NIRS) was used in this study to measure muscle oxidative capacity and offers a non‐invasive technique that allows the measurement of oxygenation changes in the microvasculature (e.g., arterioles, capillaries, and venules) using near‐infrared light (700–10,000 nm) that can penetrate several centimeters (cm) into biological tissues (4–5 cm in muscles) (Orcioli‐Silva et al., 2024; Subhas & Smith, 2011). Oxygenated and deoxygenated hemoglobin absorb light at different wavelengths in the near‐infrared range, which allows photodetectors to monitor these changes in the human body. Accordingly, the percentage of oxyhemoglobin relative to total hemoglobin can be used to calculate the tissue saturation (also known as tissue saturation index, TSI%), which reveals the local tissue oxygenation status (Orcioli‐Silva et al., 2024).

NIRS is widely used to assess oxidative metabolism in several skeletal muscles during exercise in chronic conditions including peripheral artery disease, type 2 diabetes, chronic kidney disease, multiple sclerosis and LC (Appelman et al., 2024; Collins et al., 2012; Gildea et al., 2021; Tuesta et al., 2022). Changes in TSI% have been reported in response to both treadmill and cycle exercise protocols as well as arterial and venous occlusions (Appelman et al., 2024; Kuge et al., 2005; Mezzani et al., 2013; Tuesta et al., 2022). Appelman et al. reported lower power output at the gas exchange threshold in people living with LC compared with controls and observed a reduction in peripheral oxygen extraction. Using NIRS of the vastus lateralis muscle, changes of muscle deoxygenation relative to maximum during incremental cycling until task failure indicated less peripheral oxygen extraction in people with LC. CPET across 2 days has also revealed dysfunctional physiological limitations at submaximal thresholds in people living with LC that indicate impaired oxygen delivery and extraction (Gattoni et al., 2025; Thomas, Kudiersky, et al., 2025). To date, no study has conducted 2‐day CPET in conjunction with NIRS and/or at submaximal intensities to confirm these limitations, included objective measures to mitigate the risk of PEM, and the comparison of a control group.

Accordingly, this study aimed to: (1) determine any between‐day and/or group differences in CPET outcomes, (2) assess within‐day changes of TSI%, and (3) examine any between‐day changes of TSI% in people living with LC.

## 2 MATERIALS AND METHODS

### 2.1 Ethical approval

This single‐center, cross‐sectional, observational study received both NHS (IRAS ID: 313936) and institutional (ETH2324‐1808) research ethics approval. All participants provided written informed consent before their involvement in the study, and the study conformed to the standards set by Good Clinical Practice and by the Declaration of Helsinki (Version 2024).

### 2.2 Participants

#### 2.2.1 Recruitment

Convenience sampling methods, supported by a social media campaign, word of mouth, and an existing database of LC participants held by the University of Derby, were utilized for participant recruitment for both LC and control cohorts.

#### 2.2.2 Screening and eligibility

Eligibility for the LC cohort was established via telephone screening and required a confirmed COVID‐19 history, a clinical LC diagnosis consistent with the WHO definition (WHO, 2022), and the absence of moderate–severe PEM, assessed using the DePaul Symptom Questionnaire (DSQ; Cotler et al., 2018). Moderate–severe PEM was defined as reporting a 3 or 4 for symptom frequency and severity on the DSQ. Control participants were free from confounding comorbidities (e.g., chronic obstructive pulmonary disease, coronary artery disease, diabetes, etc.). Eligibility criteria that applied to both cohorts included having sufficient English language comprehension and cognitive ability to provide informed consent and be able to complete all study assessments as per the study protocol. Due to the exercise component of the study, standard CPET safety criteria were applied (Liguori & American College of Sports Medicine, 2020), which excluded individuals with conditions such as unstable angina, uncontrolled hypertension (resting systolic blood pressure [SBP] >180 mmHg, or resting diastolic blood pressure [DBP] >110 mmHg), severe orthopedic conditions, recent embolism, and other significant metabolic or cardiovascular conditions.

Screening and eligibility were assessed by CT and confirmed by MF. Thirteen people living with LC were ineligible for participation at the recruitment stage for the following reasons: high‐risk of PEM (*n* = 9), a serious medical condition (*n* = 2), or were unable to commit to the study requirements (*n* = 2). Following confirmation of eligibility, 46 individuals with confirmed LC (*n* = 32 female) were invited to complete a series of three face‐to‐face visits over 10 days. Ten control participants completed two submaximal CPET visits across 2 days separated by 24‐h.

### 2.3 Study visits

#### 2.3.1 Baseline assessment (visit 1)

A thorough description of baseline procedures and patient‐reported outcome measures is detailed elsewhere (Thomas, Kudiersky, et al., 2025); however, briefly, LC participants attended three laboratory visits following confirmation of eligibility. Baseline data collection consisted of a full demographic profile (age, sex, smoking history, and past medical history), detailed acute COVID‐19 history (number of confirmed infections, vaccinations, hospital admissions, acute symptoms, retrospective assessment of performance status), and LC information (diagnosis, symptoms, current access to treatments/services). Symptom severity and frequency, as well as impact upon functional status and quality of life, were assessed via several questionnaires, including the post‐COVID‐19 functional status scale (Klok et al., 2020), symptom score (Thomas, Kudiersky, et al., 2025), EuroQol 5‐Dimension 5‐Level (Herdman et al., 2011), Fatigue Assessment Scale (Michielsen et al., 2003), Modified Fatigue Impact Scale (Larson, 2013), and Medical Research Council Dyspnoea Scale (Bestall et al., 1999). Physiological observations were also measured at baseline and are presented in Table 2.

**FIGURE 1:**
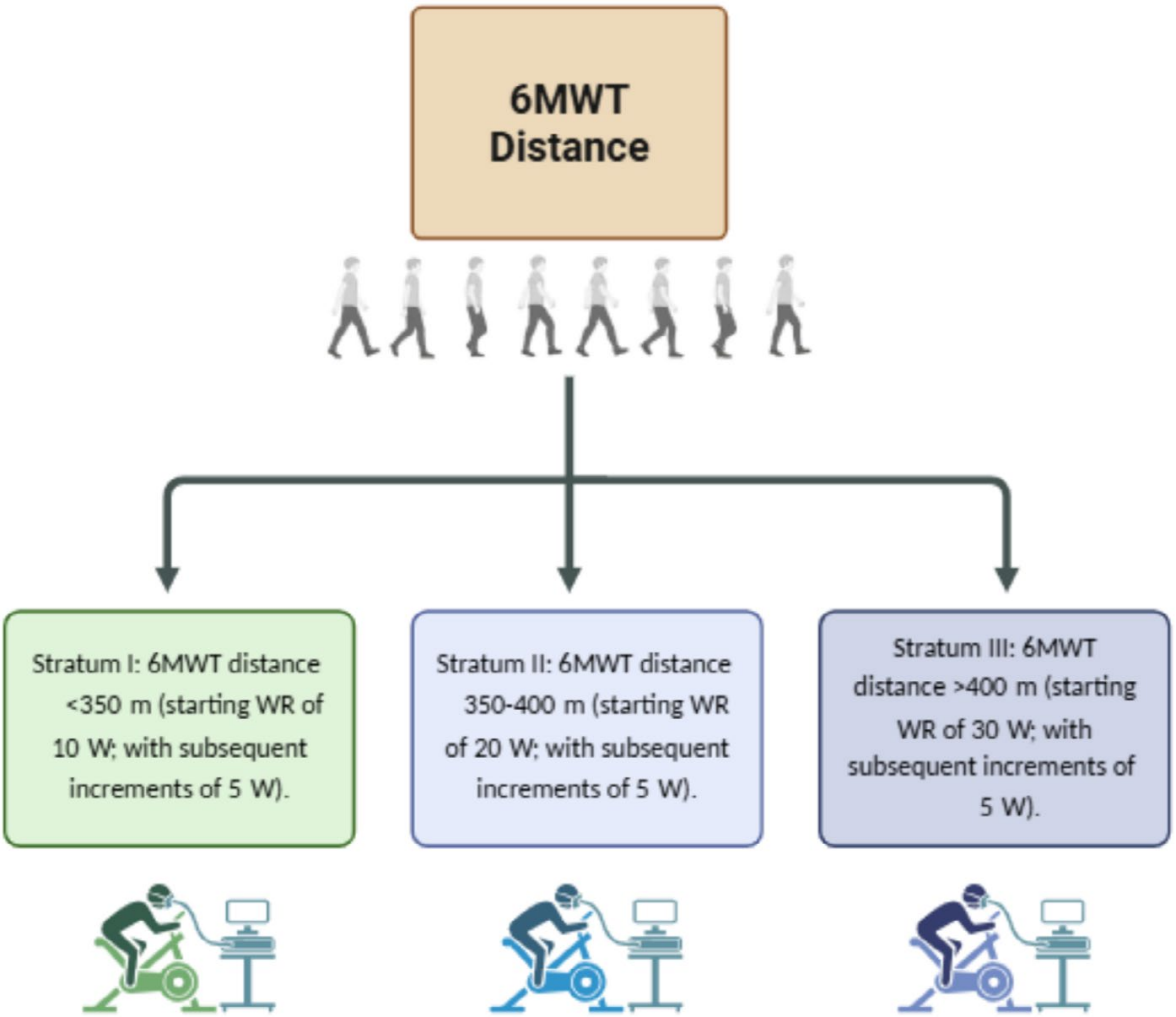
Submaximal cycling protocol selection determined by total 6‐min walk test (6MWT) distance. By C. Thomas created in BioRender (https://biorender.com/shortURL) is licensed under CC BY 4.0.

Functional status was assessed via the 6‐min walk test (6MWT) during the baseline visit and was conducted according to American Thoracic Society guidelines (Holland et al., 2014). The distance covered during the 6MWT was used to determine the starting work rate (WR) of an adapted CPET test for the LC cohort (strata outline below). All control participants completed Stratum III of the CPET protocol. In accordance with pilot data (Thomas, Kudiersky, et al., 2025), the starting WR was based on 6MWT distance for the LC cohort, as depicted in Figure 1.

#### 2.3.2 Cardiopulmonary exercise test (CPET) protocol (visits 2 and 3)

The protocol for the CPET session is detailed fully elsewhere (Thomas, Kudiersky, et al., 2025). Briefly, participants completed two submaximal CPET sessions, separated by 24‐h, using an incremental exercise ramp test on a friction‐loaded cycle ergometer (Monark 894E Ergomedic Peak Bike, Monark, Varberg, Sweden) following the American Thoracic Society guidelines (Liguori & American College of Sports Medicine, 2020). The protocol began with a 3‐min rest period, followed by a maximum of 12 min of cycle exercise at ~60 revolutions per minute (rpm) delivered via a stepwise incremental protocol. Test termination criteria included, but were not limited to, a consistent drop in cadence below 60 rpm despite encouragement and participant volition, whereby symptom exacerbation was beyond the tolerable limits of the participant.

### 2.4 Adverse event reporting

Participants were asked at each study visit to report any adverse events following any of the study visits; this included contacting the study team directly within the 7 days post completion of study activities. Adverse events were reported and investigated by appropriate clinical personnel (TB).

### 2.5 Near‐infrared spectroscopy (NIRS)

NIRS‐derived signals for oxyhaemoglobin [O_2_Hb], deoxyhaemoglobin [HHb], total hemoglobin (THB = [O_2_Hb] + [HHb]) and tissue saturation index (TSI% = [O_2_Hb]/[THB] × 100) were continuously monitored pre‐ and post‐exercise during seated rest and unloaded and loaded cycle exercise with an Artinis Portalite MKII device (Artinis Medical Systems BV, Zetten, Netherlands). The NIRS optode was placed on the area of greatest muscle mass on the left gastrocnemius muscle for each participant and was covered/secured using bandages to avoid displacement of the device and to shield against contamination of ambient light. Upon completion of the test on day one, the placement of the optode was marked using an anatomical marker pen so that the optode could be placed in the same position on day two.

### 2.6 Data processing

CPET data were reviewed for completeness by the test site coordinator (CT). Raw gas analysis data were transformed from breath‐by‐breath to the middle 5‐of‐7 breath and 30‐s moving averages to reduce variability and identify data outliers or missing data. Definitions for VT1 and the respiratory compensation point (VT2) are defined in previous work (Thomas, Kudiersky, et al., 2025; Thomas, Nunes, et al., 2025). VT1 and VT2 were determined using the V‐slope method of the middle 5‐of‐7 breaths data. Standardized American College of Sports Medicine guidelines (Liguori & American College of Sports Medicine, 2020) were used to make exercise threshold decisions by the test site coordinator (CT) with 10% checked by an additional author (RA). Any uncertainty on exercise threshold decisions was resolved through discussion between MF, CT, and RA where consensus originally had not been reached. A detailed account of CPET data processing and analysis can be found in our previous work (Thomas, Kudiersky, et al., 2025). NIRS data were exported at a frequency of 100 Hz and processed using Microsoft Excel. TSI% during seated rest periods before and after cycle‐exercise (5‐min), as well as loaded (1‐min stages) and unloaded pedaling periods (30 s), were calculated.

### 2.7 Statistical analysis

#### 2.7.1 Demographics

Data are presented as mean ± standard deviation. Normal distribution was assessed using Shapiro‐Wilks tests. Between‐group differences for parametric data were assessed using Welch's *t*‐tests; nonparametric data were assessed using Mann–Whitney tests in GraphPad Prism (Version 10, IBM Corp., Armonk, NY, USA) with a set α‐level of 0.05.

#### 2.7.2 CPET

Data are presented as mean ± standard deviation. Normal distribution was assessed using Shapiro‐Wilks tests. For between‐day, same groups analysis, parametric data were assessed using two‐tailed paired samples *t*‐tests; nonparametric data were assessed using two‐tailed Wilcoxon Signed Rank tests in GraphPad Prism with a set α‐level of 0.05 (Bonferroni corrected 0.013). For within‐day, between‐group analysis, parametric data were assessed using Welch's *t*‐tests; nonparametric data were assessed using Mann–Whitney tests. Cohen's d was used to calculate the effect size of parametric paired samples *t*‐tests with thresholds set at 0.2 = small, 0.5 = medium, and 0.8 = large where significance was reported. To calculate the effect size value of Wilcoxon's signed‐rank *t*‐tests, the formula Difference between sums of ranks/Total of sums of ranks was implemented with thresholds set at 0.1 = small, 0.3 = medium, and 0.5 = large where significance was reported.

#### 2.7.3 NIRS

Data are presented as mean ± standard deviation. Normal distribution checks were assessed with Shapiro–Wilk tests. Parametric data for between‐day differences were assessed using a two‐tailed paired samples *t*‐test; nonparametric data were assessed using two‐tailed Wilcoxon's signed rank test in GraphPad Prism with a set α‐level of 0.05. Parametric data for within‐day differences for the LC cohort were assessed using mixed‐effects analysis with Tukey multiple corrections applied. Parametric data for the control cohort were assessed using repeated‐measures one‐way ANOVA with Tukey multiple corrections applied. Non‐parametric data from day 1 for LC participants were assessed using Friedman's test with Dunn's multiple corrections applied. Mixed‐effects analysis could not be performed for this dataset due to violations of normality, meaning participants with missing data were removed from this analysis. Reasons for missing data included not being able to complete the test due to symptom exacerbation (*n* = 6) and error with device signal during measures (*n* = 3). Sphericity was not assumed for all within‐day analyses with Geisser–Greenhouse corrections applied. No between‐group comparisons were made for TSI% in this study due to the variability of probe location between different participants. Participants will vary as to the area and volume of greatest calf muscle mass, as well as the amount of adipose tissue surrounding this area, which has previously been identified as a confounding factor for NIRS measurement (Niemeijer et al., 2017).

## 3 RESULTS

LC and Control participant demographics are presented in Tables 1 and 2. No severe adverse or adverse events were reported following CPET on either protocol day. Six LC participants (13%) were unable to complete the full 12‐min protocol on both day 1 and day 2. Reasons for test termination were due to symptom exacerbation (breathlessness (*n* = 7), fatigue/heaviness in legs (*n* = 4), and dizziness (*n* = 1)). All control participants completed the full 12‐min protocol on both days.

**TABLE 1:** Participant demographics.

	Long COVID (*n* = 45)	Controls (*n* = 10)	*p* Value
Age (years)	52 ± 11	33 ± 13	<0.001^a^
Sex *n* (%)			
Male	14 (30%)	6 (60%)	0.142
Female	32 (70%)	4 (40%)	
Height (cm)	167 ± 10	172 ± 10	0.117
Weight (kg)	78 ± 17	78 ± 10	0.851
Body mass index (kg/m^2^)	28 ± 6	26 ± 2	0.078

^a^ Denotes significant with 0.05 alpha level.

**TABLE 2:** Additional Long COVID demographic, symptom, functional, and physiological status information.

Participant characteristics (*n* = 46)	
Vaccinated *n* (%)	
Yes	46 (100%)
No	0 (0%)
One dose	2 (4%)
Two doses	3 (7%)
Three doses	16 (35%)
>Three doses	24 (52%)
Unknown	1 (2%)^a^
Comorbidities *n* (%)	
Yes	35 (76%)
No	11 (24%)
Endocrine/diabetes	6 (13%)
Renal	4 (9%)
Cardiovascular	19 (41%)
Gastrointestinal/Liver	13 (28%)
Neurological/cerebrovascular	17 (37%)
Malignancy including hematological	2 (4%)
Respiratory	15 (33%)
Rheumatological	7 (15%)
Psychological	5 (9%)
Other	9 (20%)
PROMs^a^	
PCFS (AU)	3 ± 1
EQ‐5D‐5L utility score (AU)	0.74 ± 0.15
EQ‐5D‐5L VAS (AU)	57 ± 15
Symptom Score (AU)	21 ± 9
FAS (AU)	31 ± 7
MFIS (AU)	52 ± 13
MRC dyspnoea (AU)	3 ± 1
Cognitive function (AU)	27 ± 2
Baseline physiological measures	
Systolic blood pressure (mmHg)	130 ± 19
Diastolic blood pressure (mmHg)	86 ± 11
Heart rate (bpm)	74 ± 13
SpO_2_ (%)	98 ± 1
Baseline functional status^a^	
FEV1 (Liters)	2.78 ± 0.77
FVC (Liters)	3.49 ± 0.99
FEV1/FVC (AU)	81 ± 6
PEF (L•min^−1^)	449 ± 114
MIP cmH_2_O	94 ± 33
MEP cmH_2_O	130 ± 43

^a^ Denotes data available for 45 participants. PROMs denote patient reported outcome measures. PCFS denotes Post‐COVID‐19 Functional Status Scale. EQ‐5D‐5L VAS denotes EuroQol 5‐Dimension 5‐Level visual analogue score. FAS denotes Fatigue Assessment Scale. MFIS denotes Modified Fatigue Impact Scale. MRC denotes Medical Research Council. SpO_2_ denotes peripheral oxygen saturation. FEV1 denotes forced expiratory volume in 1 s. FVC denotes forced vital capacity. PEF denotes peak expiratory flow. MIP and MEP denote maximal inspiratory and expiratory pressure, respectively.

Cardiopulmonary measures at rest, VT1, and iso‐time peak for CPET day 1 and day 2 for the LC group are presented in Table 3. Cardiopulmonary measures at rest and iso‐time peak for CPET day 1 and day 2 for the control group (CON) are presented in Table 4. No VT1 data for CON is presented in Table 3 as only two CON participants (separate participants on each day) had an identifiable VT1. Workload at iso‐time peak was defined in this study as the work rates that were matched within participants for the total duration of cycle exercise (comparison of the shortest duration reached of the two datasets) (Nicolò et al., 2014).

**TABLE 3:** Cardiopulmonary measures at rest, VT1, and iso‐time peak for CPET day one and two for long COVID participants.

*n* = 46 people living with Long COVID (mean ± standard deviation)												
	Rest				VT1 (*n* = 24)				Iso‐time peak			
	Day 1	Day 2	*p* Value	ES	Day 1	Day 2	*p* Value	ES	Day 1	Day 2	*p* Value	ES
V̇O_2_ (L•min^−1^)	0.31 ± 0.06	0.32 ± 0.06	0.143	−0.220	0.71 ± 0.14	0.65 ± 0.11	0.002^b^	0.737	1.33 ± 0.25	1.33 ± 0.22	0.864	0.025
V̇O_2_ (mL•kg^−1^•min^−1^)	3.93 ± 0.87	4.03 ± 0.96	0.219	0.210	9.7 ± 1.9	8.9 ± 1.9	0.003^b^	−0.680	17.4 ± 3.8	17.4 ± 4.0	0.509	−0.114
V̇O_2__HR (mL/b)	4.49 ± 1.14	4.51 ± 1.12	0.820	−0.040	8.3 ± 2.4	7.5 ± 1.9	<0.001^b^	−0.747	11.1 ± 2.8	11.0 ± 3.0	0.302	−0.177
HR (bpm)	71 ± 13	73 ± 14	0.005^b^	0.471	88 ± 12	89 ± 13	0.828	0.054	125 ± 25	125 ± 25	0.952	−0.011
Workload (Watts)	—	—	—	—	27 ± 12	21 ± 13	0.038^a^	−0.680	73 ± 15	73 ± 15	1.000	0.000
V̇E/V̇O_2_ (AU)	24.6 ± 3.8	25.2 ± 3.0	0.192	0.223	22.0 ± 1.9	22.1 ± 1.8	0.584	0.133	30.5 ± 6.0	30.5 ± 4.9	0.880	−0.027
V̇E/V̇CO_2_ (AU)	29.3 ± 4.2	29.7 ± 4.0	0.488	0.119	28.1 ± 2.7	28.2 ± 2.6	0.877	0.040	30.8 ± 5.1	30.9 ± 4.4	0.880	0.027
RER (AU)	0.84 ± 0.06	0.85 ± 0.05	0.099	0.280	0.78 ± 0.04	0.79 ± 0.05	0.833	0.053	0.99 ± 0.06	0.99 ± 0.06	0.745	−0.056
V̇E (L•min^−1^)	9.8 ± 2.4	10.4 ± 2.3	0.010^a^	0.434	18.4 ± 2.6	17.4 ± 3.0	0.018^a^	−0.547	45.0 ± 13.4	44.7 ± 10.8	0.737	−0.058
BF (breaths/min)	15 ± 4	16 ± 4	0.007^b^	0.454	19 ± 5	20 ± 6	0.040^a^	0.480	28 ± 9	28 ± 9	0.983	0.005
V̇CO_2_ (L•min^−1^)	0.26 ± 0.05	0.27 ± 0.05	0.036^a^	−0.319	0.56 ± 0.12	0.51 ± 0.10	0.006^b^	0.623	1.32 ± 0.28	1.31 ± 0.25	0.750	0.047
P_ET_O_2_ (mmHg)	109 ± 5	110 ± 4	0.005^b^	0.469	101 ± 5	102 ± 5	0.037^a^	0.487	111 ± 6	111 ± 5	0.483	0.120
P_ET_CO_2_ (mmHg)	33.2 ± 3.3	32.5 ± 3.2	0.023^a^	0.348	37.6 ± 4.4	36.5 ± 3.2	0.015^a^	0.535	36.4 ± 5.3	36.0 ± 4.3	0.214	0.186

*Note*: ES denotes the effect size. VT1 denotes the first ventilatory threshold identified across CPET days. V̇O_2_ denotes oxygen uptake. V̇O_2__HR denotes oxygen pulse. VE/V̇O_2_ denotes the ventilatory equivalent for oxygen. VE/V̇CO_2_ denotes the ventilatory equivalent for carbon dioxide. RER denotes respiratory exchange ratio. VE denotes minute ventilation. BF denotes breathing frequency. V̇CO_2_ denotes carbon dioxide production. P_ET_O_2_ and P_ET_CO_2_ denote end‐tidal oxygen and carbon dioxide, respectively.

^a^ Denotes significant with 0.05 alpha level.

^b^ Denotes significant 0.01 alpha level.

**TABLE 4:** Cardiopulmonary measures at rest and iso‐time peak for CPET day one and two for control participants.

*n* = 10 controls (mean ± standard deviation)						
	Rest			Iso‐time peak		
	Day 1	Day 2	*p* Value	Day 1	Day 2	*p* Value
V̇O_2_ (L•min^−1^)	0.32 ± 0.04	0.33 ± 0.09	0.238	1.36 ± 0.14	1.40 ± 0.16	0.367
V̇O_2_ (mL•kg^−1^•min^−1^)	4.14 ± 0.45	4.26 ± 1.01	0.640	17.7 ± 3.0	18.2 ± 2.6	0.625
V̇E/V̇O_2_ (AU)	24.4 ± 3.4	24.0 ± 4.6	0.787	25.2 ± 2.3	24.9 ± 2.6	0.513
V̇E/V̇CO_2_ (AU)	28.8 ± 3.1	29.0 ± 4.9	0.906	27.2 ± 1.3	27.0 ± 1.8	0.735
RER	0.85 ± 0.05	0.83 ± 0.06	0.207	0.93 ± 0.05	0.92 ± 0.05	0.565
V̇E (L•min^−1^)	10.1 ± 1.8	10.7 ± 3.3	0.456	37.9 ± 5.1	38.4 ± 5.7	0.565
BF (breaths/min)	15 ± 4	17 ± 3	0.161	24 ± 4	23 ± 6	0.598
V̇CO_2_ (L•min^−1^)	0.27 ± 0.04	0.27 ± 0.07	0.823	1.26 ± 0.16	1.29 ± 0.18	0.461
P_ET_O_2_ (mmHg)	111 ± 3	111 ± 4	0.625	107 ± 3	107 ± 3	0.912
P_ET_CO_2_ (mmHg)	32.9 ± 1.0	32.1 ± 1.8	0.262	39.7 ± 1.4	39.4 ± 2.0	0.659

*Note*: V̇O_2_ denotes oxygen uptake. V̇E/V̇O_2_ denotes the ventilatory equivalent for oxygen. V̇E/V̇CO_2_ denotes the ventilatory equivalent for carbon dioxide. RER denotes respiratory exchange ratio. VE denotes minute ventilation. BF denotes breathing frequency. V̇CO_2_ denotes carbon dioxide production. P_ET_O_2_ and P_ET_CO_2_ denote end‐tidal oxygen and carbon dioxide, respectively.

### 3.1 Within‐day outcomes

#### 3.1.1 Day 1

Iso‐time peak work rate was lower in LC when compared with CON on day 1 (73 ± 15 W vs. 85 ± 0 W; *p* = 0.001). Compared with CON, V̇E/V̇O_2_ (17%, 30.5 ± 6.0 AU; *p* < 0.001), V̇E/V̇CO_2_ (12%, 30.8 ± 5.1 AU; *p* < 0.05), RER (6%, 0.99 ± 0.06; *p* < 0.01), and P_ET_O_2_ (3%, 111 ± 6 mmHg; *p* < 0.05) were all higher in LC on day 1 at iso‐time peak. P_ET_CO_2_ (8%, 36.4 ± 5.3 mmHg; *p* < 0.001) was greater in CON when compared with LC at iso‐time peak. Rest blood lactate in LC was 1.3 ± 0.5 mmol/L and increased to 3.5 ± 1.6 mmol/L post‐test (*p* < 0.001). No differences were observed at rest for any CPET variables for either the LC or CON on day 1.

**FIGURE 2:**
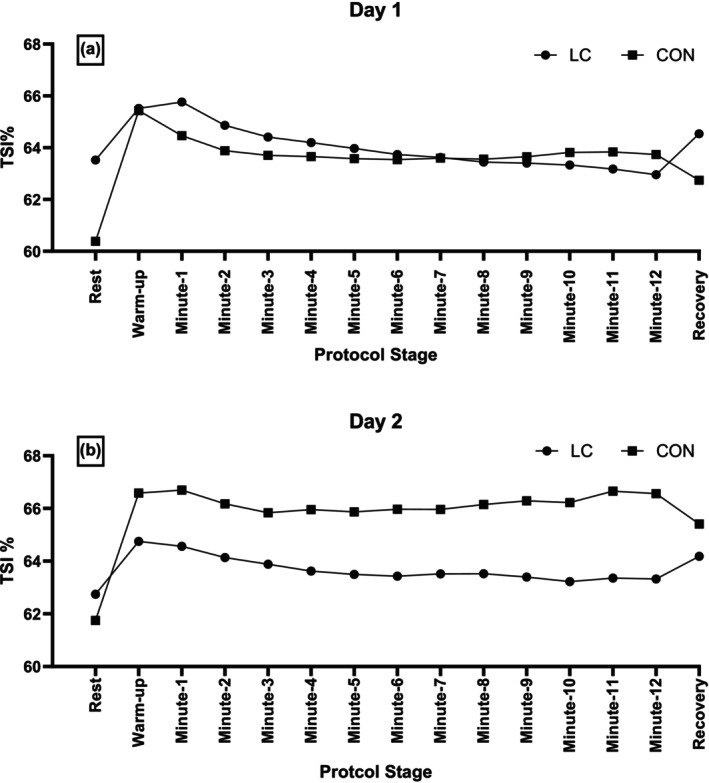
Tissue saturation index (TSI%) before, during and after submaximal cycle exercise in long COVID (LC) and Control (CON) participants on: (a) Day 1 and (b) Day 2.

Figure 2a depicts within‐day TSI% responses of LC and CON to submaximal CPET on day 1. TSI% in LC increased above rest during the unloaded pedaling warm‐up phase (*n* = 33; 64 ± 5 vs. 66 ± 5%; *p* < 0.001). TSI% remained elevated above rest up until and including the 2nd minute of cycle exercise from which point TSI% was no longer different compared to rest despite the increasing work rate and duration of exercise (*p* > 0.05). TSI% decreased gradually from minute‐9 (*p* < 0.05) to minute‐12 (*p* < 0.001) compared with the unloaded pedaling and minute‐1 of the exercise phase, indicating a return to resting values. In CON, TSI% increased above rest during the unloaded pedaling warm‐up phase on day 1 (*n* = 10; 60 ± 4 vs. 65 ± 2%; *p* < 0.05). TSI% remained elevated above rest up until the 5th minute of cycle exercise from which point TSI% was no longer different compared to rest (*p* > 0.05).

#### 3.1.2 Day 2

On day 2, iso‐time peak work rate was lower in LC when compared with CON (73 ± 15 W vs. 85 ± 0 W; *p* = 0.001). V̇E/V̇O_2_ (18%, 30.5 ± 4.9 AU; *p* < 0.001), V̇E/V̇̇CO_2_ (13%, 30.9 ± 4.4 AU; *p* < 0.01), RER (6%, 0.99 ± 0.06; *p* < 0.01), and P_ET_O_2_ (4%, 111 ± 5 mmHg; *p* < 0.05) were all higher in LC compared with CON at iso‐time peak. P_ET_CO_2_ (8%, 36.0 ± 4.3 mmHg; *p* < 0.001) was greater in CON when compared with LC on day 2 at iso‐time peak. Rest blood lactate in LC was 1.4 ± 0.6 mmol/L which increased to 3.3 ± 1.7 mmol/L post exercise (*p* < 0.001). No differences were observed at rest for any CPET variables for either the LC or CON on day 2.

Figure 2b depicts within‐day TSI% responses of LC and CON to submaximal CPET on day 2. On day 2, TSI% in LC increased above rest during the unloaded pedaling warm‐up phase (*n* = 42; 63 ± 5 vs. 65 ± 4%; *p* < 0.05). However, TSI% remained elevated above rest up until and including the 1st minute of cycle exercise, only from which point onwards TSI% was no longer different compared to rest (*p* > 0.05). On day 2, TSI% in CON increased above rest during the unloaded pedaling warm‐up phase (*n* = 10; 62 ± 5 vs. 67 ± 3%; *p* < 0.05). However, TSI% remained elevated above rest values throughout the whole duration of the exercise protocol (*p* < 0.05) and was no longer different compared to rest during the recovery phase.

### 3.2 Between‐day analysis

Compared with day 1, on day 2 heart rate (mean increase 4 ± 5%), minute ventilation (mean increase 6 ± 4%), breathing frequency (mean increase 8 ± 10%), and P_ET_O_2_ (mean increase 2 ± 8%) for LC were all increased at rest (Table 3). At VT1, V̇O_2_ (mean reduction 9 ± 20%), oxygen pulse (mean reduction 9 ± 22%), and V̇CO_2_ (mean reduction 9 ± 17%) for LC were all reduced on day 2 (Table 3). No between‐day differences were observed for CPET variables at iso‐time peak for either LC or CON. No between‐day differences were observed for any of the CPET variables at rest for CON. VT1 was not identifiable on both days for any CON participant. There were no between‐day differences in peak blood lactate at rest (*p* = 0.451) or post‐exercise (*p* = 0.087) in LC.

Table 5 shows the results recorded between days for LC and CON. No differences were observed for resting values of TSI% for LC (*n* = 41; 64 ± 5 vs. 63 ± 5%; *p* > 0.05) or CON (*n* = 10; 60 ± 4 vs. 62 ± 5%; *p* > 0.05) cohorts. No differences were observed in TSI% for those with an identifiable VT1 in LC across both days (*n* = 22; 65 ± 5 vs. 65 ± 4; *p* > 0.05). This trend continued for each of the exercise minutes and recovery period for both groups (Table 4).

**TABLE 5:** Tissue oxygenation saturation (TSI%) of the gastrocnemius muscle across 2‐day submaximal CPET in LC and control participants.

	LC				Control			
	*n*	Day 1	Day 2	*p* Value	*n*	Day 1	Day 2	*p* Value
Baseline	41	64 ± 5	63 ± 5	0.060	10	60 ± 4	62 ± 5	0.385
Warm‐up	41	66 ± 5	65 ± 4	0.322	10	65 ± 2	67 ± 3	0.267
Minute‐1	41	66 ± 5	65 ± 4	0.081	10	65 ± 3	67 ± 3	0.090
Minute‐2	41	65 ± 5	64 ± 5	0.105	10	64 ± 4	66 ± 3	0.084
Minute‐3	41	65 ± 6	64 ± 5	0.217	10	64 ± 4	66 ± 4	0.087
Minute‐4	41	64 ± 6	64 ± 6	0.140	10	64 ± 4	66 ± 4	0.066
Minute‐5	39	64 ± 6	64 ± 6	0.099	10	64 ± 4	66 ± 4	0.081
Minute‐6	39	64 ± 6	64 ± 6	0.126	10	64 ± 5	66 ± 4	0.090
Minute‐7	39	64 ± 7	64 ± 6	0.115	10	64 ± 5	66 ± 4	0.089
Minute‐8	38	64 ± 7	64 ± 6	0.124	10	64 ± 5	66 ± 4	0.089
Minute‐9	38	64 ± 7	63 ± 7	0.094	10	64 ± 5	66 ± 4	0.094
Minute‐10	36	64 ± 7	63 ± 7	0.578	10	64 ± 5	66 ± 4	0.118
Minute‐11	33	64 ± 7	63 ± 7	0.805	10	64 ± 5	67 ± 4	0.065
Minute‐12	32	63 ± 7	63 ± 7	0.938	10	64 ± 5	67 ± 4	0.057
Recovery	39	65 ± 6	64 ± 6	0.125	10	63 ± 6	65 ± 6	0.090

## 4 DISCUSSION

The primary finding of this study is that individuals with LC exhibit a diminished capacity to sustain oxygen delivery during submaximal cycle exercise. There was no change in TSI% response between days, indicating that oxygen transport and delivery are unlikely to be the only factors driving the observed decline in CPET performance in people with LC. This data underlines the need to increase the mechanistic understanding of contributing and interacting pathologies to develop future management, rehabilitative, and treatment strategies for those living with LC.

### 4.1 Ventilatory complications

The present study revealed notable between‐group differences in ventilatory pattern. Specifically, LC participants demonstrated elevated V̇E/V̇O2_2_, V̇E/V̇CO_2_, RER, and P_ET_O_2_, alongside reduced P_ET_CO_2_ at iso‐time peak, compared with controls. These findings align with Aparisi et al. who report increased ventilatory inefficiency, characterized by higher V̇E/V̇CO_2_ and lower P_ET_CO_2_ at the anaerobic threshold (AT) in previously hospitalized COVID‐19 patients with persistent dyspnoea compared with asymptomatic controls (Aparisi et al., 2021). Known differences have been reported between those people with LC who had been hospitalized with a SARS‐CoV‐2 infection and those who were not (Pérez‐González et al., 2022), but it appears there may be some interesting overlap between these groups (e.g., inspiratory muscle strength) (Gach et al., 2024; Hennigs et al., 2022; Nagel et al., 2022). The observed increase in P_ET_O_2_ further suggests dysfunctional breathing and/or a mismatch between ventilation and perfusion in the lungs, supporting the need for future research to investigate these areas in more detail. Notably, unlike previous findings at the AT (Aparisi et al., 2021), the higher iso‐time peak RER in the LC cohort indicates impaired energy substrate utilization for the given absolute exercise intensity. Nevertheless, additional mechanisms within the oxygen delivery and utilization pathway (e.g., impaired cardiac output, microvascular dysfunction, altered muscle fiber composition, and mitochondrial dysfunction) are likely contributing to the early shift toward anaerobic metabolism observed during submaximal exercise in individuals with Long COVID.

### 4.2 Impaired microcirculation

SARS‐CoV‐2 infection is well‐documented to cause increased platelet activation and damage to the microcirculation, including degradation of the endothelial glycocalyx layer (Wu et al., 2024). The glycocalyx plays a critical role in regulating microvascular resistance and capillary hemodynamics; thus, COVID‐19 related damage could contribute to tissue hypoxia (Østergaard, 2021). During acute infection, elevated levels of angiopoietin‐2 (ANG‐2) and vascular endothelial growth factors (VEGFs) have been observed, indicating endothelial activation and vascular instability (Vassiliou et al., 2023). These biomarkers increase vascular permeability by promoting leakage across the alveolar‐capillary barrier, contributing to pulmonary oedema and impaired gas exchange (Bellani et al., 2016; Vassiliou et al., 2023; Wu et al., 2024). Furthermore, these elevated biomarkers cause microvascular dysfunction through a reduction in capillary integrity and oxygen delivery and promote a prothrombotic state as endothelial activation can trigger clot formation, leading to microthrombi and ventilation‐perfusion mismatch (Kell & Pretorius, 2022). These changes could amplify inflammatory signaling, perpetuating tissue damage and fibrosis risk, causing the chronic sequelae that is LC. Patel et al. reported that increased ANG‐1 and P‐selectin (P‐SEL) levels could classify LC cases with 96% accuracy, implicating endothelial dysfunction as a potential pathology (Patel et al., 2022). Although the present study did not include blood biomarker analyses, reduced TSI% data suggest impaired oxygen delivery that may be consistent with the hypothesis of capillary damage and increased microvascular resistance in those living with LC. Future research is warranted to examine this proposed relationship and progress this mechanistic knowledge base.

The presence of fibrinaloid microclots could also impair oxygen transport. These heterogeneous, amyloid‐state biomarkers are composed of inflammatory proteins, fibrinogen chains, and antifibrinolytic molecules, which render them highly resistant to proteolytic breakdown (Kell & Pretorius, 2017). As a result, they have been proposed to persist in circulation, obstructing microvascular function, impairing gas exchange, and potentially contributing to the early onset of anaerobic metabolism observed in individuals with LC in this study and elsewhere (Kell et al., 2022; Pretorius et al., 2021; Thomas, Nunes, et al., 2025). Previous work from our lab indicates that microclots may fragment into smaller aggregates following submaximal CPET (Thomas, Nunes, et al., 2025), which may enter and/or impede the microvasculature. This could reduce perfusion, potentially exacerbate post‐exercise dysfunction, and impair physiological function. However, Appelman et al. found that after maximal CPET designed to induce PEM in a LC cohort, substantial amyloid‐containing deposits were not found within capillaries, but rather within the extracellular matrix when compared to controls. This suggests that while microclots may play a role in the development of PEM, their distribution, movement characteristics, underlying mechanisms, and outcomes remain unclear and warrant further investigation.

### 4.3 Impaired oxygen extraction

Invasive CPET (iCPET) offers insight into identifying localized physiological dysfunction and research using this method has shown distinct endotypes of LC (Risbano, 2025; Risbano et al., 2023); for example, preload failure and impaired systemic oxygen extraction. Combining real‐time blood assessment of arterial and venous blood with measures of whole‐body oxygen consumption allows researchers to determine whether oxygen delivery or extraction is impaired within exercising participants (Leitner et al., 2024). Kahn et al. (2024) observed that for 75% of LC participants with exertional intolerance who took part in iCPET, peak systemic oxygen extraction was impaired. This study also observed that this impairment was no different between those who have undertaken supervised rehabilitation, reaffirming the need for targeted management, rehabilitative, and therapeutic strategies for those living with LC. Although the approach in the present study lacks the specificity to localize physiological dysfunction, the reduction in oxygen pulse at VT1 in the LC cohort on day 2 compared with day 1, as well as the elevated P_ET_O_2_ compared with the control cohort, supports the notion that oxygen is not extracted appropriately in the LC cohort. Combining NIRS with techniques such as venous occlusion induced by a pneumatic cuff would allow for the measurement of peripheral fractional oxygen extraction (Wolfsberger et al., 2022). This would provide greater insight into this dysfunction and allow for better specificity during noninvasive CPET.

### 4.4 Mitochondrial dysfunction

SARS‐CoV‐2 exploits host cell mitochondria for viral replication, leading to structural damage characterized by altered mitochondrial morphology, size, and number (Molnar et al., 2024). These disruptions impair normal mitochondrial functioning resulting in reduced energy production, and mitochondrial dysfunction is linked to symptoms of LC (Molnar et al., 2024). For example, differently regulated mitochondrial proteins and structural abnormalities, including mitochondrial swelling and disrupted cristae, have been reported (Molnar et al., 2024; Peppercorn et al., 2023). Additionally, Appelman et al. proposed that reduced maximal mitochondrial respiration and decreased mitochondrial content may be an important consideration in the pathophysiology of PEM. This may be one of many factors that caused elevated blood lactate levels >3.0 mMol/L post‐CPET on both days from the LC cohort in the present study, as seen with myalgic encephalomyelitis/chronic fatigue syndrome (ME/CFS) patients across 2‐day CPET (Lien et al., 2019). Davenport et al. observed similar bioenergetic patterns between LC and ME/CFS through 2‐day CPET (e.g., significant reductions in V̇O_2_ and workload at the ventilatory anaerobic threshold in both LC and ME/CFS compared to non‐disabled controls) (Davenport et al., 2026). However, it is important to note that, although the underlying pathophysiology of both conditions remains unclear, the observed similarities beyond the work by Davenport et al. are largely based on commonly reported symptoms rather than confirmed shared mechanisms. Detailed investigations, including skeletal muscle biopsies, cardiopulmonary imaging, invasive hemodynamic assessments, mitochondrial functional assays, and metabolomic profiling could provide further insight into whether mitochondrial limitations and other peripheral or central factors contribute to the progressive impairment observed during 2‐day CPET in individuals with LC and ME/CFS.

### 4.5 Chronic inflammation

Biomarkers with proinflammatory properties have been commonly identified as part of diagnostic LC reviews (Espin et al., 2023; Lai et al., 2023; Thomas et al., 2024); however, the evidence often varies between participants as no established profile of biomarkers have been identified. It has been suggested that inflammatory subgroups may exist; for example, Talla et al. identified two distinct inflammatory subtypes of LC in their cohort (Talla et al., 2023). This may indicate that subgrouping may be possible for several other pathologies (e.g., endothelial dysfunction, microclotting, and mitochondrial dysfunction); however, given the mechanistic overlap that has been postulated between hypotheses (e.g., inflammation has been associated with viral persistence and microclot breakdown; Pretorius et al., 2021; Proal et al., 2025) these cannot be mutually exclusive. Heightened inflammation has also been correlated with metabolic dysfunction (Aid et al., 2025), and such profiling may optimize patient profiling and improve referral toward therapeutic targets that are more likely to be helpful for those individuals (e.g., antiviral therapy may be beneficial for individuals with persistently heightened inflammatory profiles). CPET and NIRS could be helpful within the context of this pathology; however, targeted blood biomarker profiling of the inflammatory subtypes identified by Talla et al. is required concurrently to support this approach.

### 4.6 Dysautonomia

Dysautonomia and chronotropic intolerance (e.g., abnormally low heart rate reaction) are common in people living with LC (Mustonen et al., 2024). Using the criteria applied by Mustonen et al., identifying dysautonomia using the protocol in the present study could be calculated with the addition of heart rate recovery data. Meanwhile, alternative criteria of chronotropic intolerance that do not require maximal testing could also be applied (e.g., degree of change in heart rate during the 6MWT) (Provencher et al., 2006). Impaired endothelial and microvascular function indicated by the findings of the present study has been linked to cardiovascular symptoms of LC, parallel to postural orthostatic tachycardia syndrome or as a part of broader cardiovascular dysautonomia (Fedorowski et al., 2023; Oikonomou et al., 2022). Other proposed mechanisms include abnormal sympathetic activity and excess circulating catecholamines, peripheral sympathetic noradrenergic denervation, which is responsible for venous pooling (Vernino et al., 2021). Venous blood pooling has previously been assessed by NIRS of the gastrocnemius muscle (Stone et al., 2016; Truijen et al., 2012) and may offer an additional and potentially more suitable, simple, and inexpensive assessment of dysautonomia for people living with LC.

### 4.7 Study limitations

The variability in probe placement and unaccounted differences in adipose tissue thickness prevented valid between‐group comparisons of TSI% in this study. Increased adipose tissue can distort NIRS signals, leading to an overestimation of TSI%. This occurs because the signal becomes more reflective of the adipose tissue, which typically has a higher TSI% compared to skeletal muscle due to its lower metabolic rate (Niemeijer et al., 2017). However, probe placement location between days was kept identical using an anatomical marker pen on day 1, which allowed for reliable comparisons between day for the same participant. Additionally, the use of continuous‐wave NIRS and the selection of the gastrocnemius muscle rather than the vastus lateralis employed in previous studies introduce further considerations regarding signal quality and interpretation. This study did not include biomarker analyses or additional assessment techniques to confirm endothelial damage or mitochondrial dysfunction, both of which have been hypothesized to impair oxygen delivery and contribute to abnormal energy metabolism and PESE/PEM. Age matching was not applied to recruitment in this study, with the LC group significantly older than the CON group. It is possible that the worsened ventilatory responses observed for LC during incremental CPET may have been impacted by this factor (e.g., V̇E/V̇CO_2_ slope increases with age; Phillips et al., 2020), and this uncertainty could be resolved by recruiting similarly aged CON groups in future work.

### 4.8 Practical implications and future recommendations

The present study makes use of submaximal exercise approaches, which identified VT1 in over half of the LC cohort on both days. This response may serve as a practical diagnostic tool for clinicians supporting individuals with LC. Nonetheless, given the overlap of these physiological responses with other conditions such as ME/CFS and the contraindication of such testing for those with moderate‐to‐severe PEM, further diagnostic measures are needed to accurately identify, manage, and treat affected individuals. Future research integrating muscle biopsies and endothelial and mitochondrial biomarkers alongside the current methodology could yield valuable mechanistic insights. However, such investigations must carefully balance the burden placed on participants with the cost‐effectiveness and potential impact of findings. Traditionally, the vastus lateralis has been more commonly used as opposed to the gastrocnemius for NIRS measurements during cycling assessments as this muscle is more heavily involved in cycling tasks (Iskra & Paravlić, 2025). During pilot testing with LC volunteers, we encountered several issues when using the vastus lateralis (e.g., sensor displacement, time to affix the device, and participant comfort) which added to the participant burden in addition to the additional time required for questionnaire completion and cognitive assessment. Accordingly, we chose the gastrocnemius as a convenient, well‐tolerated and previously used muscle of interest to complement the additional measurements. Future use of vastus lateralis NIRS measurements with LC participants during CPET would further LC mechanistic understanding and enable greater comparison to the broader literature. Research designs may seek to reduce participant burden by isolating this measure and remove additional time constraints or complete them remotely on separate days (e.g., questionnaire completion). Furthermore, the use of NIRS for assessment in those living with LC has potential applications for the metabolic assessment of other regions of the body (e.g., brain) (Murkin & Arango, 2009; Simonson & Piantadosi, 1996). Future work may apply this technology without exercising the individual, allowing for inclusion of those who present with a greater risk of more severe PEM, which may provide pathophysiological insight driving their symptom presentation.

## 5 CONCLUSION

This study demonstrates that individuals with LC who had a low risk of moderate‐to‐severe PEM exhibited an inability to sustain oxygen delivery during submaximal cycle exercise. These findings could partially explain the early onset of anaerobic energy utilization observed within CPETs. Future research should aim to utilize NIRS, CPET, and additional biomarkers to better understand the role mitochondrial dysfunction, chronic immune activation, and dysautonomia have for LC pathophysiology to identify possible diagnostic, rehabilitative, and therapeutic options.

## AUTHOR CONTRIBUTIONS

**Callum Thomas:** Conceptualization; data curation; formal analysis; funding acquisition; investigation; methodology; project administration; visualization. **Ruth E. Ashton:** Conceptualization; data curation; formal analysis; funding acquisition; investigation; methodology; supervision. **Rebecca Owen:** Data curation; formal analysis; funding acquisition; investigation; project administration. **Ethan McNeil‐Angopa:** Data curation; formal analysis; funding acquisition; investigation. **Jack Carr:** Data curation; formal analysis; funding acquisition; investigation. **Thomas Bewick:** Conceptualization; formal analysis; funding acquisition; investigation. **Mark A. Faghy:** Conceptualization; data curation; formal analysis; funding acquisition; investigation; methodology; resources; supervision; visualization.

## FUNDING INFORMATION

This work received a funding grant from Gilead Sciences (IN‐UK‐983‐6080).

## CONFLICT OF INTEREST STATEMENT

No potential competing interest was reported by the authors.

## ETHICS STATEMENT

NHS (IRAS ID: 313936) and institutional (ETH2324‐1808) research ethics was approved for this study.

## Data Availability

Anonymized data can be provided upon request.
